# Combining two-dimensional gel electrophoresis and metabolomic data in support of dry-season survival in the two main species of the malarial mosquito *Anopheles gambiae*^☆^

**DOI:** 10.1016/j.dib.2015.08.031

**Published:** 2015-09-07

**Authors:** K. Hidalgo, K. Mouline, W. Mamai, N. Foucreau, K.R. Dabiré, A. Bouchereau, F. Simard, D. Renault

**Affiliations:** a Université de Rennes 1, UMR CNRS 6553 Ecobio, Campus de Beaulieu, 263 Avenue du Général Leclerc, CS 74205 35042 Rennes Cedex, France; b Institut de Recherche pour le Développement (IRD), UMR IRD 224-CNRS 5290-Université de Montpellier MIVEGEC, 911 Avenue Agropolis, BP 64501, 34394 Montpellier cedex 5, France; c Institut de Recherche en Sciences de la Santé (IRSS), Direction Régionale de l’Ouest (DRO), 399 Avenue de la Liberté 01, BP 545 Bobo-Dioulasso, Burkina Faso; d Université Claude Bernard Lyon 1, UMR CNRS 5023 LEHNA, 43 Bd du 11 Novembre 1918, 69622 Villeurbanne Cedex, France; e Université de Rennes 1, UMR INRA IGEPP, Campus de Beaulieu, 263 Avenue du Général Leclerc, CS 74205, 35042 Rennes Cedex, France

**Keywords:** 2D electrophoresis, Amino acid, Metabolomic, Mosquito, Proteomic, Sugar

## Abstract

In dry savannahs of West-Africa, the malarial mosquitoes of the *Anopheles gambiae sensu stricto* complex annually survive the harsh desiccating conditions of the dry season. However, the physiological and biochemical mechanisms underlying how these mosquitoes survive such desiccating conditions are still undefined, and controversial. In this context, we provide the first work examining both proteomic and metabolomic changes in the two molecular forms of *A. gambiae s.s* (M and S forms) experimentally exposed to the rainy and dry season conditions as they experience in the field. Protein abundances of the mosquitoes were measured using a two-dimensional fluorescence difference gel electrophoresis (2D DIGE) coupled with a matrix-assisted laser desorption/ionisation-time of flight (MALDI-TOF) and tandem mass spectrometry (MS) for protein identification. These assays were conducted by Applied Biomics (http://www.appliedbiomics.com, Applied Biomics, Inc. Hayward, CA, USA), and the mass spectrometry proteomics data have been deposited to the ProteomeXchange Consortium (http://proteomecentral.proteomexchange.org) via the PRIDE partner repository with the dataset identifier PXD000294. The metabolomic analysis was conducted using both Acquity UPLC^®^ system (for amino acid identification), and a gas-chromatography-mass spectrometry platform (for sugars identification). Metabolomic fingerprintings were assessed in the University of Rennes 1, UMR CNRS 6553 EcoBio (France). A detailed interpretation of the obtained data can be found in Hidalgo et al. (2014) [1] (Journal of Insect Physiology (2014)).

Specifications Table [please fill in right-hand column of the table below]

**Table:** 

Subject area	*Biology*
More specific subject area	*Ecophysiology; Proteomics; Medical Entomology*
Type of data	*Tables & barplots*
How data was acquired	*Proteomic data were assessed using a two-dimensional fluorescence difference gel electrophoresis (2D DIGE) coupled with a matrix-assisted laser desorption/ionisation-time of flight (MALDI-TOF) and tandem mass spectrometry (MS)*
*Metabolomic data were assessed using a gas-chromatography-mass spectrometry (GC–MS) and an Acquity UPLC*® *system (Waters Corporation, Milford, MA, USA) platforms*
Data format	*Raw, analyzed*
Experimental factors	*We compared the protein and metabolites (amino acids, polyols, sugars) in two malarial mosquito species form the* Anopheles gambiae *species complex (formerly known as the M and S molecular forms). The two species were reared under climatic conditions mimicking the rainy and dry season conditions as they experience naturally.*
Experimental features	*Two-dimensional fluorescence difference gel electrophoresis (2D DIGE); matrix-assisted laser desorption/ionisation-time of flight (MALDI-TOF) and tandem mass spectrometry (MS); gas-chromatography-mass spectrometry (GC–MS) and Acquity UPLC*^®^*platforms*
Data source location	*Both M and S mosquitoes were collected in Burkina-Faso (West Africa) in human dwellings of Bama (*11°23*'N,* 04°24*′W) and Soumousso (*11°01'N*,* 04°02*′W), respectively. The metabolomic data were collected at the University of Rennes 1, UMR CNRS 6553 EcoBio (France). The proteomic data were obtained from Applied Biomics (USA).*
Data accessibility	*The mass spectrometry proteomics data have been deposited to the ProteomeXchange Consortium* (http://proteomecentral.proteomexchange.org) *via the PRIDE partner repository with the dataset identifier PXD000294.*

Value of the data

• The use of 2D gel electrophoresis comparisons identified interesting changes in several protein abundances from one season to the next in mosquitoes;
• The comparisons of mosquito's metabolic fingerprints highlighted specific changes in their metabolite contents (amino acids and sugars) from one season to the next;
• By combining both 2D gel electrophoresis and metabolic fingerprint comparisons our approach improves the identification of physiological and biochemical changes in mosquitoes.

**Data, experimental design, materials and methods**

## 1. Data

Mosquitoes from the *Anopheles gambiae s.l.* complex (both M and S molecular forms of *A. gambiae*) are widespread in West Africa where they seasonally experienced the harsh desiccating conditions of the dry season. How these mosquitoes survive the dry season and are engaged in exponential population growth as soon as the rainy season starts is still undefined and very controversial [2,3]. This ability to survive the dry season suggests high physiological plasticity in mosquitoes. In order to highlight the general physiological changes involved in mosquitoes at the onset of the dry season, we compared protein abundance and metabolic fingerprints in the two molecular forms of *A. gambiae* exposed to the climatic conditions of the rainy and the early dry season.

The M and S molecular forms of *A. gambiae* were recently described as two different species, named Anopheles *coluzzii* and *A. gambiae*, respectively [4,5]. Here, we are still referring to the M and S molecular forms, as in our published paper (Hidalgoet al. [1]).

## 2. Experimental design

Mosquitoes were reared from eggs to adults into four programmable climatic chambers (Sanyo MLR 315 H, Sanyo Electric Co., Osaka, Japan). Two climatic chambers were used to reproduce the climatic conditions of the rainy season, and two others to reproduce those of the dry season. Climatic conditions were programmed using the temperature and humidity cycles recorded in south-western Burkina-Faso (11°23′N, 04°24′W) with a Vantage Pro2 weather monitoring station (Weatherlink; Davis Instruments, Hayward, CA, U.S.A.). Climatic conditions were recorded in August 2010 (rainy season) and December 2010 (onset of the dry season), then hourly averaged to design 12 step cycles required to programme as closely as possible the natural daily climatic variations inside the climatic chambers [6]. Conditions inside the climatic chambers were tightly monitored throughout experiments using MicroLog Pro monitors (EC750, Davis Instruments, Hayward, CA, U.S.A.).

## 3. Proteomic assays

For each mosquito species (M and S molecular forms) and rearing conditions (rainy and dry season), proteomic samples consisted of a pool of 30 teneral (1 h-old adult) female mosquitoes. Samples were sent to the proteomics department of Applied Biomics (http://www.appliedbiomics.com, Applied Biomics, Inc. Hayward, CA, USA) for proteomic assays as described below.

### 3.1. Protein extraction and adjustment of the concentration level

A 2-D cell-lysis buffer (30 mM Tris–HCl, pH 8.8, containing 7 M urea, 2 M thiourea, and 4% 3-[(3-Cholamidopropyl) dimethylammonio]-1 propanesulfonate [CHAPS]) was used to extract the proteins in each sample of 30 pooled females. Then, the protein concentration of each sample was measured using a Bio-Rad protein assay method, and adjusted to 5 mg.ml^−1^ using the 2-D cell-lysis buffer.

### 3.2. Samples labelling

For both species, we used the CyDye DIGE Fluor Cy3 (red) and Cy5 (green) saturation dyes (4 mL, 2 mM) to label samples from the rainy and the dry season rearing conditions, respectively. A Cy2 dye, corresponding to an internal standard for the normalisation of protein abundances, was also used to label a 50:50 mixture of the two rearing conditions samples. The three CyDye DIGE Fluors were incubated with corresponding samples for 30 min on ice, under dark conditions, before adding a 1 µL volume of 10 mM lysine. A 2× 2-D sample buffer (8 M urea, 4% CHAPS, 20 mg/mL DTT, 2% pharmalytes and a trace amount of bromophenol blue) was used to terminate the reaction, and the samples were stored at −80 °C until further use for 2D DIGE assays.

### 3.3. Two-dimensional fluorescence difference gel electrophoresis (2D DIGE)

First, 30 µg of both Cy3- and Cy5-labelled samples were mixed with a DeStreak^TM^ rehydration solution (0.5% pharmalyte pH 3–10 in a 250 µL final volume; GE Healthcare, Waukesha, WI, USA). Then, samples were loaded onto an isoelectric focusing (IEF) strip (pH 4–10 linear range; GE Healthcare) with an Ettan IPGPhore II at standard conditions. After IEF, the strips were incubated in an equilibration buffer (50 mM Tris–HCl, pH 8.8, containing 6 M urea, 30% glycerol, 2% SDS, a trace amount of bromophenol blue and 10 mg.mL^−1^ DTT) for 15 min, and rinsed in an equilibration buffer 2 (50 mM Tris–HCl, pH 8.8, containing 6 M urea, 30% glycerol, 2% SDS, a trace amount of bromophenol blue and 45 mg.mL^−1^ iodoacetamide) for 10 min.

The electrophoresis was performed at 15 °C on a 12% SDS–PAGE gel. Note that each gel contained the three Cy3, Cy5 and Cy2 labelled samples. For each mosquito species, we performed three-replicate gels to compare the effects of rearing conditions on their protein expression (*N*=6 gels in total). A seventh gel containing the two species exposed to dry conditions and their 50:50 mixture was run. In this last gel, the M molecular form was labelled with Cy5 dye, whereas the S form was labelled with Cy3 dye.

### 3.4. Image analysis

**Fig. 1:**
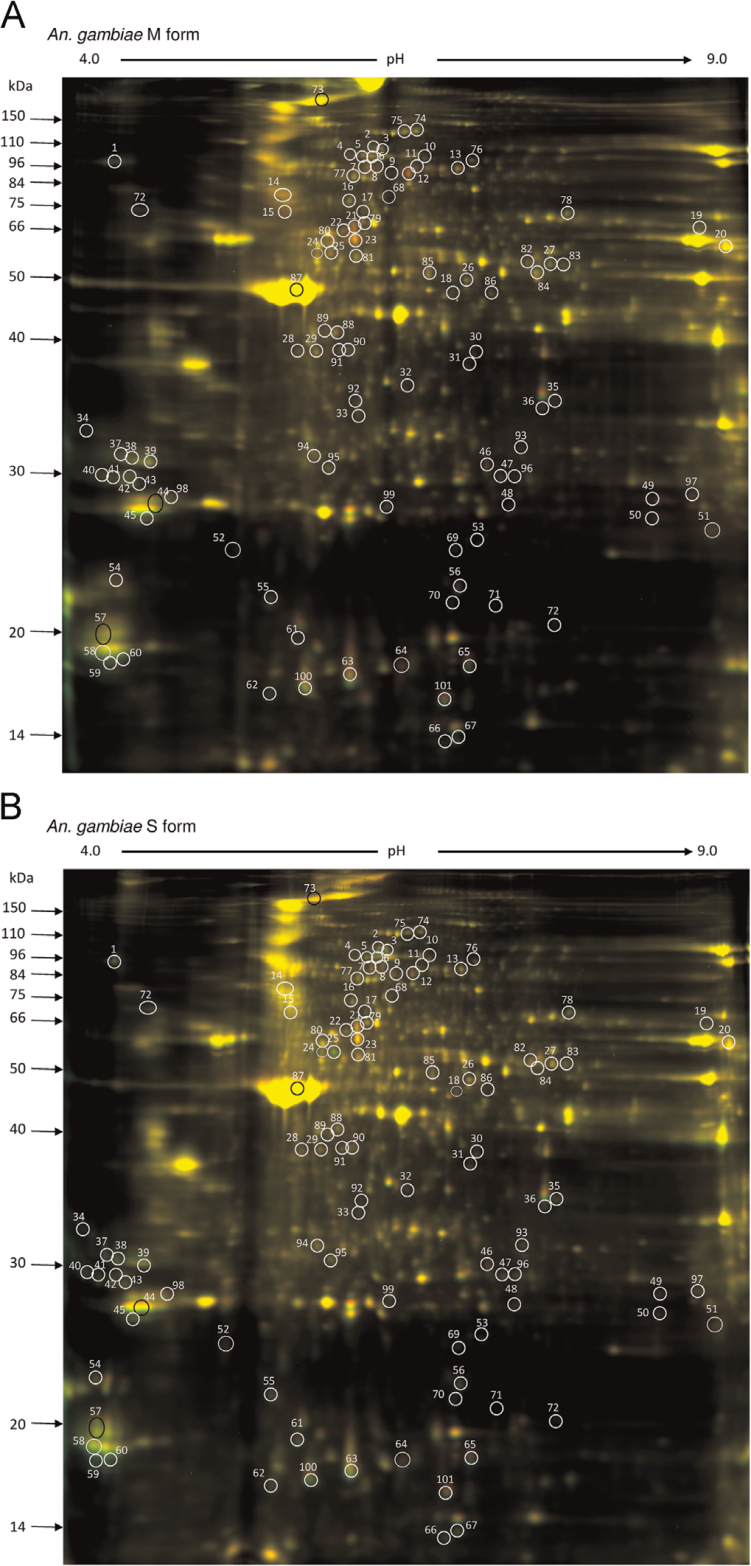
Representative image of the two-dimensional fluorescence difference gel electrophoresis (2D DIGE); *A. gambiae* M (A), *A. gambiae* S (B). Females reared under the dry conditions were labelled with Cy3 (green), whereas females reared under the rainy conditions were labeled with Cy5 (red). From Hidalgo et al. [1].

Effect of rearing conditions on protein expression in both species and differences between species were assessed by overlaying images of the seven different gels. Note that the abundance of each protein spot was normalised using the seventh gel. The resulting 2D gels were scanned using a Typhoon Trio scanner (Amersham BioSciences) with excitation and emission wavelengths for Cy2-labelled (488/520 nm), Cy3-labelled (548/560 nm) and Cy5-labelled (641/660 nm) proteins using settings that match in similar relative fluorescence intensities for the Cy3- or Cy5-labelled samples (Fig. 1). Image analysis for intensity measurements of the different protein spots was performed using ImageQuantTL and DeCyder softwares (GE Healthcare). Then, images were subjected to in-gel analysis and cross-gel analysis using DeCyder v6.5 (GE-Healthcare) with a *P*-value <0.05 according to Student’s *t*-tests. Based on these tests, we retained a total of 109 spots, corresponding to proteins with molecular masses ranging from 14 to 150 kDa, and isoelectric points between 4 and 9. These spots showed significant variation in at least one species and/or one rearing condition.

### 3.5. Protein identification using matrix-assisted laser desorption/ionisation-time of flight (MALDI-TOF) and tandem mass spectrometry (MS)

Identification of the 109 protein spots of interest was performed by Applied Biomics, Inc. Spots were picked up from the gel using an Ettan spot picker (GE Healthcare), and washed twice with 25 mM ammonium bicarbonate and 50% acetonitrile to remove the remaining dyes. Then, proteins were digested in-gel at 37 °C using a modified porcine trypsin protease (Trypsin Gold, Promega) according the method described in Rosenfeld et al. [7]. The digested tryptic peptides were desalted using Zip-tip C18 (Millipore, Billerica, MA), mixed with 0.5 µL of α-cyano-4-hydroxycinnamic acid (CHCA) matrix and spotted into wells of a MALDI plate. Mass spectra of the peptides in each digested spot were obtained using a MALDI-TOF (MS) and a TOF/TOF (tandem MS/MS) equipment (AB Sciex). The MALDI-TOF mass spectra data were acquired in reflectron positive ion mode, averaging 2000 laser shots per spectrum. The TOF/TOF tandem MS fragmentation spectra were acquired for each sample, averaging 2000 laser shots per fragmentation spectrum on each of the 5-10 most abundant ions present in each sample (excluding trypsin autolytic peptides and other known background ions). Both the peptide mass and the associated fragmentation spectra were submitted for database search using GPS Explorer software version 3.5 (Applied Biosystems) equipped with the MASCOT search engine (http://www.matrixscience.com, Matrix science). This submission allows the identification of proteins from the National Center for Biotechnology Information non-redundant protein sequence database (NCBInr), restricted to the *A. gambiae* species complex. The searches were performed without constraining the protein molecular weight or isoelectric point, with carbamidomethylation and oxidation as variable modifications, and with one missed cleavage allowed in the search parameters. The highest protein scoring hit with a protein score confidence interval over 95% from the database search for each 2D gel spot was accepted as positive identification.

Based on an average difference of at least 1.3-fold (absolute value), only 39 spots were selected, which corresponded to 29 distinct proteins, for further identification. The ratio of the 39 spot expressions between the species and/or rearing conditions was obtained from in-gel analysis using DeCyder. Table 1 shows the identification of the 39 spots retained in *A. gambiae* based on this method.

### 3.6. Data analysis

Average-fold differences of the 39 spot abundances were first normalised (Logarithmic abundance of the protein contents from each 2D DIGE gel), then calculated for the four following comparisons: (i) rainy *vs.* dry condition in *A. gambiae* M; (ii) rainy *vs.* dry condition in *A. gambiae* S; (iii) *A. gambiae* M *vs. A. gambiae* S when exposed to rainy conditions; and (iv) *A. gambiae* M *vs. A. gambiae* S when exposed to dry conditions. For each ratio, differences in spot abundance were assessed using Student’s *t*-tests on individual spots (*P*-value <0.05).

**Fig. 2:**
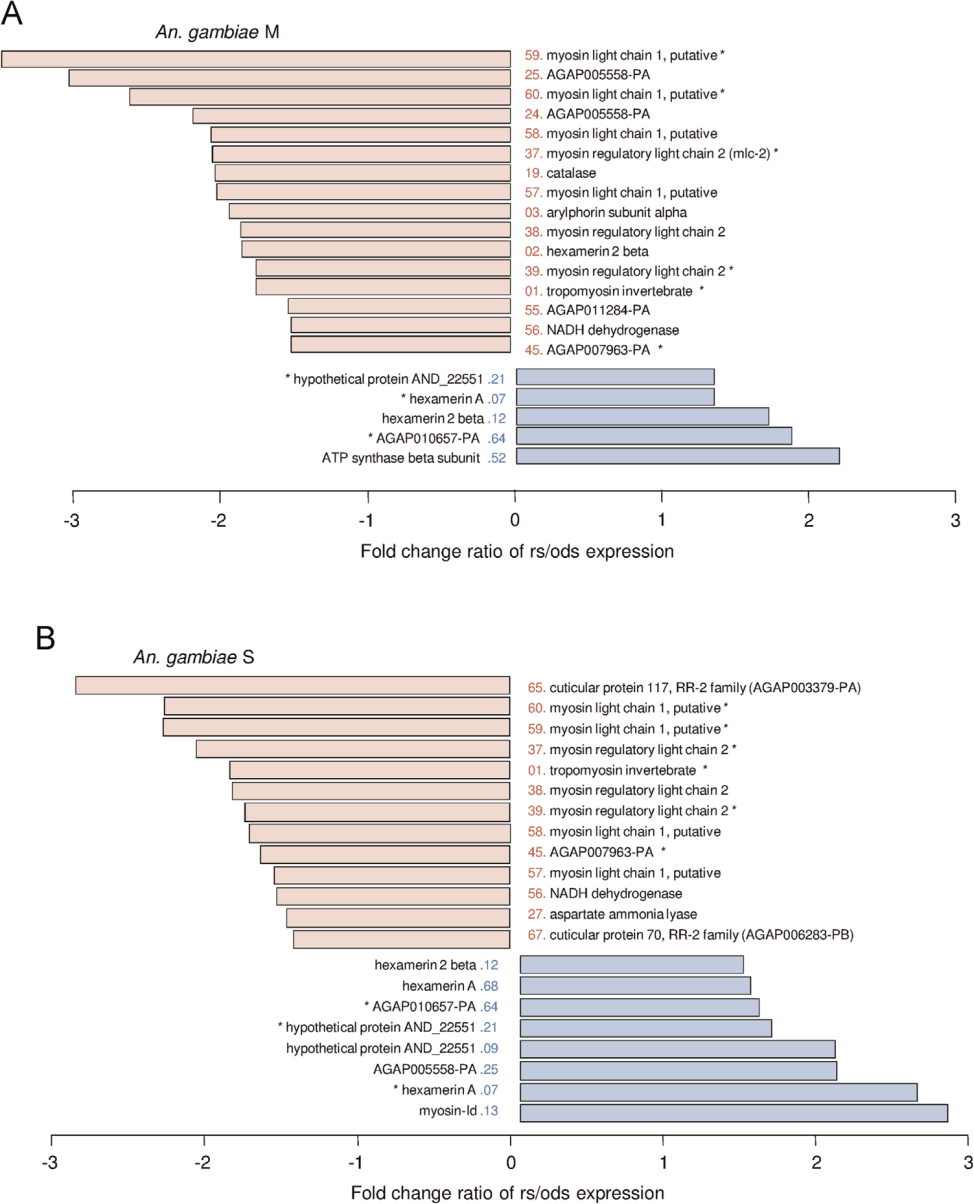
Differential fold-change of the 21 selected spot proteins between rainy and dry conditions in 1-h old females of *Anopheles gambiae* M (A) and S (B). Blue bars are positive values that represent spots that were more abundant in rainy conditions, whereas red bars are negative values that represent spots that were more abundant in dry conditions. Spots with “⁎” represent the nine protein spots exhibiting the same pattern of variations in both species. From Hidalgo et al. [1].

Accordingly, we observed that 15 proteins exhibited significant variations between the two rearing conditions in 1-h old females *A. gambiae* M, among which 10 were more abundant when females grew under the dry conditions (Fig. 2A). In contrast, 14 proteins showed significant variations between the two rearing conditions in 1-h old females *A. gambiae* S, among which 8 were more abundant when females grew under dry conditions (Fig. 2B). Overall, nine proteins exhibited the same pattern of variations in both species: six proteins increased under dry conditions, whereas the three others decreased under these conditions (Fig. 2A and B).

**Fig. 3:**
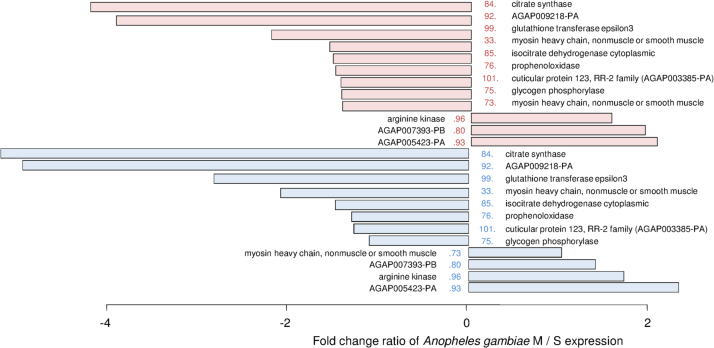
Differential fold-change of the 24 selected spot proteins between M and S forms reared under rainy (blue bars) and dry (red bars) conditions. Positive values represent spots that were more abundant in *Anopheles gambiae* M, whereas negative values represent spots that were more abundant in *Anopheles gambiae* S. Six proteins were more abundant in females *A. gambiae* S, whatever the experimental conditions, among which prophenoloxidase (spot #76), citrate synthase (spot #84), isocitrate dehydrogenase (spot #85), and glutathione transferase (spot #99) can be mentioned. The RR-2 cuticular protein 123 (spot #101) was also significantly more abundant under the dry conditions (Student *t*-test, *P*<0.001), although its fold change was only 1.27. In addition, a phosphorylase (spot #75) was more abundant in *A. gambiae* S compared to *A. gambiae* M when the females were exposed to the dry conditions, but no differential expression was found under rainy conditions. Arginine kinase (spot #96) was always more abundant in *A. gambiae* M, whatever the experimental conditions. From Hidalgo et al. [1].

Finally, 24 proteins showed significant variation between species whatever the rearing conditions. Six of the 24 proteins were always more abundant in females *A. gambiae* S (Fig. 3). For details about data interpretation and conclusion please report to Hidalgo et al. [1].

## 4. Metabolomic assays: amino acid and sugar concentrations

Metabolomic fingerprinting was conducted on both 1 h-old and 24 h-old adult females. For each mosquito species (M and S molecular forms) and rearing conditions (rainy and dry season), metabolomic samples consisted of a pool of five–six female specimens, so that the dry mass of each sample reached at least 1 mg. Five to nine replicates were used for the different experimental conditions.

### 4.1. Extraction of the metabolites

Samples were first freeze-dried (Lyovac™ GT3) for 72 h, before being weighed using a micro-balance (Mettler Toledo GmbH^©^, Greinfense, Switzerland, *d*=1 µg). Two 3 mm tungsten beads and a 1000 µL volume of methanol-chloroform (2:1) solution were added to each sample. Then, each sample was shaken at 30 Hz for 1.5 min (Retsch™ MM301, bead-beating, Retsch GbmH, Haan, Germany). We added a 500 μL volume of ultrapure water. Samples were vortexed for homogenisation and centrifuged at 4000*g* for 10 min at 4 °C. Finally, we collected and transferred a 600 µL aliquot of the upper aqueous phase (containing amino acids, polyols and sugars) into clean microtubes. The 600 µL aliquot was vacuum-dried (Speed Vac Concentrator, Genevac Ltd., Ipswitch, England), and a volume of 600 µL of ultrapure water was added to the residual.

### 4.2. Identification and quantification of amino acids

Twenty µL of each sample previously extracted were diluted with 60 µL of ultrapure water. Then, a 5 μL volume of each diluted sample was transferred into a new microtube for derivatization. Derivatization was conducted according to the AccQTag ultra derivatization kit (Waters Corporation, Milford, MA, USA) procedures. After reconstituting the AccQTag Ultra Reagent Powder, we prepared a 100 pmol/µl calibration standard solution (Acide Hydrolase Standard). Then, a 10 µl volume of the calibration standard solution and a 20 µl volume of AccQTag Ultra Reagent were added to each sample. Samples were vortexed and heated for 10 min at 55 °C.

After derivatization, 1 µL of each sample was transferred in an Acquity UPLC^®^ system (Waters Corporation, Milford, MA, USA) equipped with an Acquity UPLC® BEH C18 1.7 μm 2.1×100 mm^2^ column heated at 55 °C, as described by Renaultet al. [8]. The derivatized amino acids were detected at 260 nm using a photo diode-array detector. Peaks were identified according to their retention time compared with a list of 22 amino acid commercial standards (Table 2). Concentration was calculated by comparison of each amino acid peak area with those of the individual external standards.

### 4.3. Identification and quantification of sugars

A 50 µL volume of each sample previously extracted was transferred into a clean glass vial and vacuum-dried. Then, residuals were resuspended in 50 µL of freshly prepared methoxyamine hydrochloride (Sigma-Aldrich, St. Louis, MO, USA) in pyridine (20 mg/mL). Samples were incubated under orbital shaking at 30 °C for 90 min. Following incubation, 50 μl volume of N-methyl-N-(trimethylsilyl) trifluoroacetamide (MSTFA) were added for derivatization. Derivatization was conducted overnight at 37 °C.

To identify and quantify sugars, we used gas chromatography coupled with mass spectrometry (GC–MS). The GC–MS system consisted of a TriPlus autosampler, a Trace GC Ultra chromatograph and a Trace DSQII quadrupole mass spectrometer (Thermo Fischer Scientific Inc, Waltham, MA, USA). A 1 µl volume of the derivatized sample was injected into the GC–MS system using the split mode (25:1). The injector temperature was held at 260 °C. We programmed the GC–MS in order that the oven temperature remained 4 min at 70 °C after the injection, and then increased at a rate of 5 °C/min until it reached 300 °C, where it remained for 10 min. For elution, we used a 30 m fused silica column (TR5 MS, I.D. 0.25 mm, 95% dimethyl siloxane, 5% phenyl polysilphenylene-siloxane) and helium as the carrier gas at a rate of 1 mL/min.

Detection of each sugar was conducted by mass spectrometry using the electronic impact (EI) method. Briefly, the temperature of the ion source and mass spectrometry transfer line was set at 260 °C. All of the samples were run under the SIM mode (electron energy: −70 eV). Each sugar peak was annotated using both mass spectra (two specific ions), and the retention index specific to each compound. Randomised sample and standard sequences were established for the sample injection. Chromatograms were deconvoluted using the XCalibur v2.0.7 software (Thermo Fisher Scientific, San Iose, CA, USA). Standard samples consisted of the pure reference compounds (arabinose, fructose, galactose, glucose, ribose and trehalose) at 100, 200, 300, 500 and 1000 µM concentrations. Sugars concentrations were thus quantified using the quadratic calibration curves for each pure reference compound.

### 4.4. Data analysis

#### 4.4.1. Amino acids

Among the 22 amino acids we listed in our external standards, 21 were correctly detected and quantified (*i.e.* cysteine did not provide reliable measurements, as this amino acid is unstable and is quickly transformed into cystine during derivatization). Significance of amino acid fingerprintings among species and rearing conditions was assessed using multivariate discriminant analyses and MANOVAs. Because false discovery rate can be induced by multiple comparisons, we used a Benjamini–Hochberg procedure to adjust all *P*-values.

**Fig. 4:**
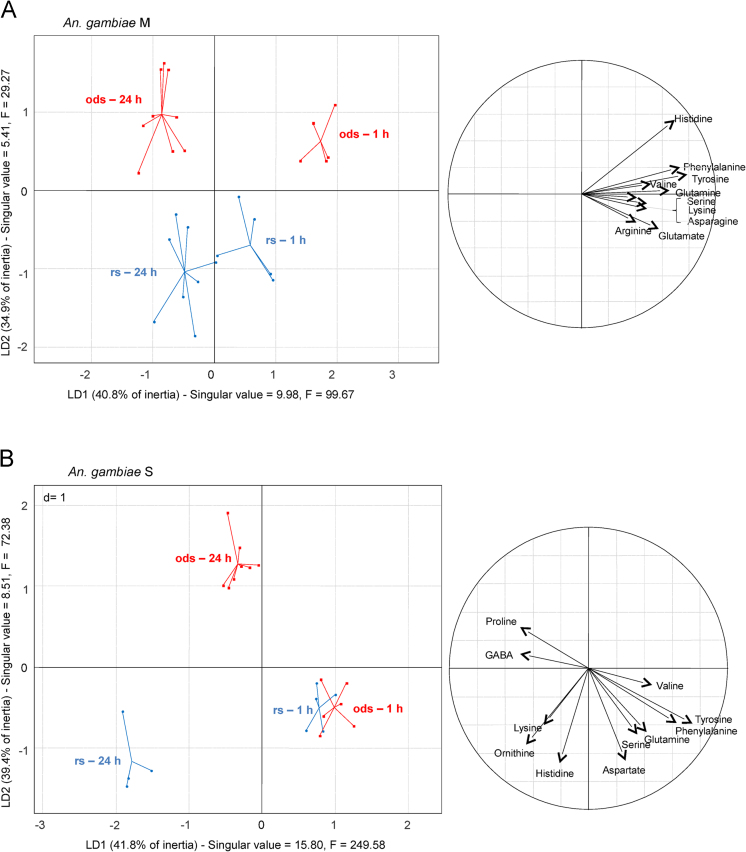
Left: Sample projections onto the first LDA discriminant plane for female *Anopheles gambiae* M (28 samples, A) and S (23 samples, B) species reared under dry (red squares) and rainy (blue dots) season conditions. Singular values refer to the ratio of the between-class and within-class inertia. Right: correlation circles depict the normalised relation (from −1 to 1) between each amino acid and LDA axes. From Hidalgo et al. [1].

Comparisons of the amino acid fingerprints of each experimental modality showed significant differences in the concentration of at least one amino acid between the two species, their ageing and their rearing conditions. Of particular interest, both species and especially M form, accumulated phenylalanine, tryptophan, tyrosine, and valine 1  h after emergence (Fig. 4A and B). S form specimens did not show fingerprint variation according to the rearing conditions 1 h after emergence, but significant changes were observed 24 h after emergence, with proline being accumulated under dry conditions (Fig. 4B). For details about data interpretation and conclusion, please report to Hidalgo et al. [1].

#### 4.4.2. Sugars

Among the six sugars listed in our reference standard, only galactose and glucose were reliably quantified in our mosquito samples. Indeed, arabinose, fructose, ribose and trehalose were below the quantification limit of the equipment used. Significance of the galactose and glucose concentrations variation among species and rearing conditions was assessed using Kruskal–Wallis tests, followed by a Bonferroni procedure resulting in a decreased threshold of significance from *P*-value <0.05 to *P*-value <0.01.

**Fig. 5:**
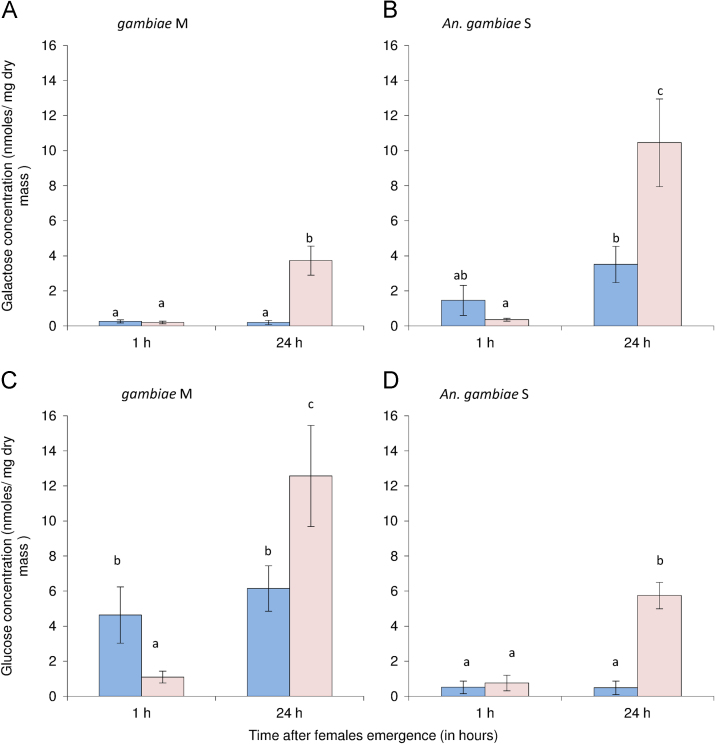
Mean (± s.e.) galactose (A and B) and glucose (C and D) levels (in *n *moles mg^−1^ dry mass) in 1-h and 24-h old females of *Anopheles gambiae* M (A and C) and S (B and D). Blue bars correspond to the females reared under rainy season conditions and light red bars correspond to females reared under dry season conditions Letters above the bars report significant differences after Bonferroni correction to account for multiple tests (*P*<0.01). From Hidalgo et al. [1].

Accordingly, both galactose and glucose concentrations were significantly higher 24 hours after emergence in the two species when mosquitoes were reared under the dry conditions (Fig. 5A*–*D). Moreover, glucose concentration was lower in M form specimens reared under the dry conditions (Fig. 5B and C). For details about data interpretation and conclusion please report to Hidalgo et al. [1].

## Acknowledgements

These assays were supported by the Agence Nationale de la Recherche through grant ANR-08-MIEN-006 to FS. The authors would like to thank Moundai Tchonfienet, Sougrinoma Zoungrana and Boubakar Nikiema (IRSS, Bobo-Dioulasso) for their help in mosquito rearing and sample preparation. Authors would like to thank the PRIDE team for their support.

## Figures and Tables

**Table 1:** List of the 39 protein spots displaying differential abundance (average fold difference>1.3, absolute value; *P*<0.05) between the two rearing conditions in 1- h old female *A. gambiae* M and S, or between the two species whatever the rearing conditions. Adapted from Hidalgoet al. [1].

**Spot no.**^a^	**Protein name**	**Protein scores**^b^	**No. Of matched peptides**	**Source species**	**Accession no.**^c^	**Functions**^d^
01	tropomyosin invertebrate	318	12	*Culex quinquefasciatus*	gi|170056897	Muscle contraction
02	hexamerin 2 beta	41	2	*Aedes aegypti*	gi|157119837	Storage of amino acid
03	arylphorin subunit alpha	134	4	*Culex quinquefasciatus*	gi|170043201	Storage of amino acid / Constituent of Cuticle sclerotizing system
07	hexamerin A	284	15	*Anopheles melas*	gi|3420171	Storage of amino acid
09	hypothetical protein AND_22551	153	15	*Anopheles darlingi*	gi|312371166	Storage of amino acid
12	hexamerin 2 beta	70	4	*Aedes aegypti*	gi|157110143	Storage of amino acid
13	myosin-Id	172	5	*Culex quinquefasciatus*	gi|170029188	Muscle contraction
19	catalase	215	16	*Anopheles gambiae*	gi|118638436	Response to oxidative stresses
21	hypothetical protein AND_22551	321	12	*Anopheles darlingi*	gi|312371166	Storage of amino acid
24	AGAP005558-PA	1000	19	*Anopheles gambiae*	gi|31213235	Proteolysis
25	AGAP005558-PA	570	16	*Anopheles gambiae*	gi|31213235	Proteolysis
27	aspartate ammonia lyase	301	15	*Aedes aegypti*	gi|157118058	Tricarboxylic acid cycle
37	myosin regulatory light chain 2 (mlc-2)	336	5	*Aedes aegypti*	gi|157167683	Muscle contraction
38	myosin regulatory light chain 2 (mlc-2)	299	5	*Aedes aegypti*	gi|157167683	Muscle contraction
39	myosin regulatory light chain 2 (mlc-2)	330	5	*Aedes aegypti*	gi|157167683	Muscle contraction
45	AGAP007963-PA	601	16	*Anopheles gambiae*	gi|118789564	Muscle contraction
52	ATP synthase beta subunit	250	12	*Culex quinquefasciatus*	gi|170040305	ATP synthesis
55	AGAP011284-PA	304	11	*Anopheles gambiae*	gi|118779554	Positive regulation of translational elongation and termination
56	NADH dehydrogenase	268	6	*Culex quinquefasciatus*	gi|170037145	Respiratory chain process
57	myosin light chain 1, putative	202	4	*Aedes aegypti*	gi|157167807	Muscle contraction
58	myosin light chain 1, putative	117	4	*Aedes aegypti*	gi|157167807	Muscle contraction
59	myosin light chain 1, putative	156	5	*Aedes aegypti*	gi|157167807	Muscle contraction
60	myosin light chain 1, putative	181	5	*Aedes aegypti*	gi|157167807	Muscle contraction
64	AGAP010657-PA	165	8	*Anopheles gambiae* str. PEST	gi|158289706	Oxygen transporter activity
65	⁎cuticular protein 117, RR-2 family (AGAP003379-PA)	709	9	*Anopheles gambiae*	gi|158290052	Structural constituent of the rigid cuticle
67	⁎cuticular protein 70, RR-2 family (AGAP006283-PB)	608	9	*Anopheles gambiae*	gi|158295676	Structural constituent of the rigid cuticle
68	hexamerin A	395	26	Anopheles gambiae	gi|3420159	Storage of amino acid
33	myosin heavy chain, nonmuscle or smooth muscle	124	17	Aedes aegypti	gi|157111095	Muscle contraction
73	myosin heavy chain, nonmuscle or smooth muscle	498	36	Aedes aegypti	gi|157110721	Muscle contraction
75	glycogen phosphorylase	635	21	Aedes aegypti	gi|157108521	Glycogenolysis process
76	prophenoloxidase	955	38	Anopheles gambiae	gi|3892092	Oxidation-reduction process
80	AGAP007393-PB	570	19	Anopheles gambiae str. PEST	gi|118778070	Cell redox homoeostasis / Glycerol ether metabolic process
84	citrate synthase	58	12	Aedes aegypti	gi|157133341	Tricarboxylic acid cycle
85	isocitrate dehydrogenase cytoplasmic	283	14	Culex quinquefasciatus	gi|170028051	Tricarboxylic acid cycle
92	AGAP009218-PA	141	4	Anopheles gambiae str. PEST	gi|118791868	Proteolysis
93	AGAP005423-PA	588	11	Anopheles gambiae str. PEST	gi|118786445	Ubiquitin-dependent protein catabolic process
96	arginine kinase	503	14	Mimomyia luzonensis	gi|284159531	Transferase / Kinase
99	glutathione transferase epsilon3	358	4	Anopheles dirus	gi|74275399	Transferase
101	cuticular protein 123, RR-2 family (AGAP003385-PA)	814	9	Anopheles gambiae str. PEST	gi|158290062	Structural constituent of the rigid cuticle

a Protein scores derived from Mascot algorithm, indicating identity or extensive homology (*P*<0.05).

b Protein accession numbers from the National Center for Biotechnology Information non-redundant (NCBInr) database.

c Positive values mean more expressed in “rs” (rainy season) conditions whereas negative values mean over expressed in “ods” (onset of the dry season) conditions.

d Protein functions are checked using http://www.uniprot.org.

**Table 2:** List of the 22 standard amino acids used for metabolomic assays.

**Name**	**abbreviation**
Alanine	Ala
Arginine	Arg
Asparagine	Asn
Aspartate	Asp
Cystein	Cys
GABA	GABA
Glutamate	Glu
Glutamine	Gln
Glycine	Gly
Histidine	His
Isoleucine	Ile
Leucine	Leu
Lysine	Lys
Methionine	Met
Ornithine	Orn
Phenylalanine	Phe
Proline	Pro
Serine	Ser
Threonine	Thr
Tryptophan	Trp
Tyrosine	Tyr
Valine	Val
